# Avaliação no Mundo Real de um Stent Eluidor de Sirolimus de Hastes Ultrafinas em Pacientes com Infarto do Miocárdio com Supradesnivelamento do Segmento ST Submetidos à Intervenção Coronária Percutânea Primária (Registro INSTEMI)

**DOI:** 10.36660/abc.20220594

**Published:** 2023-05-18

**Authors:** Gustavo N. Araujo, Guilherme P. Machado, Marcia Moura, Anderson D. Silveira, Luiz Carlos Bergoli, Felipe Costa Fuchs, Sandro Cadaval Gonçalves, Rodrigo Vugman Wainstein, Pedro A. Lemos, Alexandre S. Quadros, Marco V. Wainstein

**Affiliations:** 1 Imperial Hospital de Caridade Florianópolis SC Brasil Imperial Hospital de Caridade, Florianópolis, SC – Brasil; 2 Instituto de Cardiologia de Santa Catarina São José SC Brasil Instituto de Cardiologia de Santa Catarina, São José, SC – Brasil; 3 Hospital de Clínicas de Porto Alegre Porto Alegre RS Brasil Hospital de Clínicas de Porto Alegre, Porto Alegre, RS – Brasil; 4 Instituto de Cardiologia do Rio Grande do Sul Porto Alegre RS Brasil Instituto de Cardiologia do Rio Grande do Sul, Porto Alegre, RS – Brasil; 5 Hospital Moinhos de Vento Porto Alegre RS Brasil Hospital Moinhos de Vento, Porto Alegre, RS – Brasil; 6 Universidade Federal do Rio Grande do Sul Porto Alegre RS Brasil Universidade Federal do Rio Grande do Sul, Porto Alegre, RS – Brasil; 7 Hospital Israelita Albert Einstein São Paulo SP Brasil Hospital Israelita Albert Einstein, São Paulo, SP – Brasil; 8 Instituto do Coração Universidade de São Paulo São Paulo SP Brasil Instituto do Coração (InCor), Universidade de São Paulo, São Paulo, SP – Brasil

**Keywords:** Infarto do Miocárdio, Angioplastia, Stents Farmacológicos

## Abstract

**Fundamento:**

O padrão-ouro atual dos stents farmacológicos (SF) coronários consiste em ligas metálicas com hastes mais finas e polímeros bioabsorvíveis.

**Objetivos:**

Nosso objetivo foi comparar um stent eluidor de sirolimus de hastes ultrafinas (Inspiron®) com outras plataformas de SF de terceira geração em pacientes com infarto do miocárdio com supradesnivelamento do segmento ST (IAMCSST) submetidos à intervenção coronária percutânea (ICP) primária.

**Métodos:**

Analisamos dados de um registro multicêntrico de IAMCSST de centros de referência da Região Sul do Brasil. Todos os pacientes foram submetidos à ICP primária, seja com Inspiron® ou outro SF de segunda ou terceira geração. Foi calculado pareamento por escore de propensão (PEP) para gerar grupos semelhantes (Inspiron® versus outros stents) em relação às características clínicas e do procedimento. Todos os testes de hipótese tiveram um nível de significância bilateral de 0,05.

**Resultados:**

De janeiro de 2017 a janeiro de 2021, 1.711 pacientes foram submetidos à ICP primária, e 1.417 pacientes preencheram nossos critérios de inclusão (709 pacientes no grupo Inspiron® e 708 pacientes no grupo dos outros SF de segunda ou terceira geração). Após PEP, a amostra do estudo foi composta por 706 pacientes (353 pacientes no grupo Inspiron® e 353 pacientes no grupo dos demais SF de segunda ou terceira geração). As taxas de revascularização do vaso alvo (*odds ratio* [OR] 0,52; intervalo de confiança [IC] 0,21 a 1,34; p = 0,173), trombose de stent (OR 1,00; IC 0,29 a 3,48;p = 1,000), mortalidade (*hazard ratio* 0,724; IC 0,41 a 1,27; p = 0,257) e os desfechos cardiovasculares maiores (OR 1,170; IC 0,77 a 1,77; p = 0,526) foram semelhantes entre os grupos após um acompanhamento mediano de 17 meses.

**Conclusão:**

Nossos achados mostram que o stent Inspiron® foi eficaz e seguro quando comparado a outros SF de segunda ou terceira geração em uma coorte contemporânea do mundo real de pacientes com IAMCSST submetidos à ICP primária.

**Figura Central:**
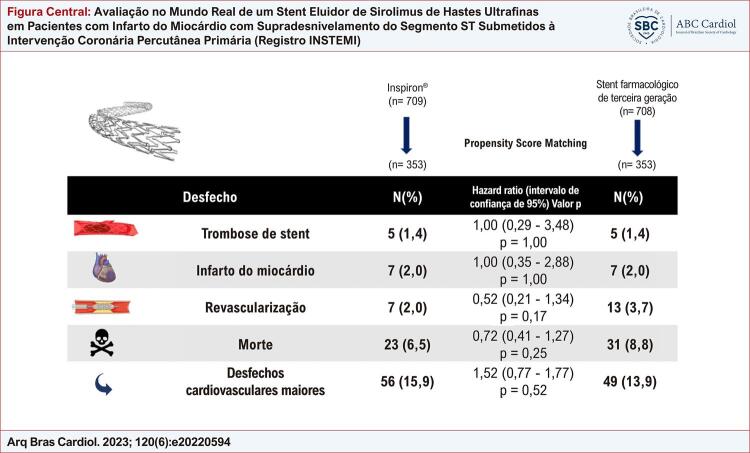
: Avaliação no Mundo Real de um Stent Eluidor de Sirolimus de Hastes Ultrafinas em Pacientes com Infarto do Miocárdio com Supradesnivelamento do Segmento ST Submetidos à Intervenção Coronária Percutânea Primária (Registro INSTEMI)

## Introdução

Os stents farmacológicos (SF) coronários estão em constante evolução, e os dispositivos mais novos devem apresentar segurança e eficácia para serem utilizados na prática diária. O infarto do miocárdio com supradesnivelamento do segmento ST (IAMCSST) é provavelmente o cenário clínico mais desafiador para comparar os SF mais recentes com aqueles já estabelecidos, devido aos riscos hospitalares e de longo prazo mais elevados de trombose de stent, infarto do miocárdio recorrente e morte.

O stent eluidor de sirolimus Inspiron® (Scitech Medical^TM^, Goiânia, Brasil) utiliza uma liga de cobalto-cromo L-605 ultrafina com uma plataforma de haste de 75 μm de espessura revestida com um polímero abluminal biodegradável. Os registros da população do tipo “*all-comers*” demonstraram segurança e excelente desempenho, com baixa taxa de eventos cardíacos adversos.^1,2^ Em um ensaio clínico randomizado comparando Inspiron® com o stent eluidor de biolimus Biomatrix Flex®, não houve diferença nos resultados em pacientes submetidos à intervenção coronária percutânea (ICP) eletiva ou urgente após cinco anos de seguimento. Além disso, não houve trombose de stent em pacientes tratados com Inspiron® durante o período do estudo.^3^

Com o uso amplamente difundido do dispositivo, são necessários dados de um número maior de pacientes em um cenário de alto risco. Nosso objetivo foi comparar o stent eluidor de sirolimus Inspiron® com outras plataformas de SF de terceira geração seguras, bem estudadas e estabelecidas em pacientes com IAMCSST submetidos à ICP primária.

## Métodos

### Desenho do estudo e seleção de pacientes

Este é um registro prospectivo, no qual incluímos pacientes consecutivos admitidos com IAMCSST e tratados com ICP primária com SF de segunda e terceira geração em dois hospitais terciários (o Hospital de Clínicas de Porto Alegre, um hospital geral, e o Instituto de Cardiologia do Rio Grande do Sul, um centro de cardiologia) na Região Sul do Brasil entre os anos de 2017 e 2021. Esta análise específica foi realizada retrospectivamente, ou seja, não havia sido pré-definida no momento em que o registro foi iniciado. O IAMCSST foi definido como dor torácica típica em repouso associada à elevação do segmento ST de pelo menos 1 mm em duas derivações contíguas no plano frontal ou de 2 mm no plano horizontal, ou como dor típica em repouso em pacientes com um bloqueio de ramo esquerdo novo ou presumivelmente novo. Os critérios de exclusão foram ausência de uso de SF de segunda e terceira geração e ausência de acompanhamento. O presente estudo foi aprovado pelo Comitê Institucional de Pesquisa e Ética de ambas as instituições e obteve consentimento informado de todos os pacientes. Os dados foram registrados prospectivamente em formulários adequados, armazenados em planilhas eletrônicas e posteriormente coletados do banco de dados.

### Aspectos do procedimento

Amostras de sangue foram coletadas por punção venosa antes do procedimento, como parte da rotina de atendimento ao paciente. Todos os pacientes foram pré-tratados com dose de ataque de ácido acetilsalicílico (300 mg) e clopidogrel (300 a 600 mg), e heparina não fracionada foi usada durante o procedimento (70 a 100 UI/kg). As estratégias técnicas de ICP e a seleção do stent foram realizadas de acordo com a escolha do operador. O fluxo coronariano antes e após o procedimento foi avaliado e descrito de acordo com os critérios Thrombolysis in Myocardial Infarction (TIMI). Os anticoagulantes foram suspensos após o término do procedimento, e a duração da terapia antiplaquetária dupla foi recomendada a critério do cardiologista. O sucesso da ICP primária foi definido como obtenção do fluxo TIMI 2 ou 3 e patência do vaso com estenose residual < 30%.

### Stents

**Figura 1:**
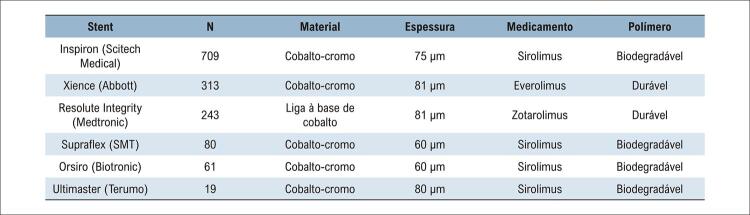
– Características dos stents farmacológicos utilizados no estudo.

Todos os pacientes incluídos receberam um SF Inspiron® ou outro de segunda ou terceira geração. A decisão de implantar qual tipo de stent foi baseada no critério do operador e na disponibilidade do centro. Além do Inspiron®, os outros SF utilizados foram Xience (laboratórios Abbott, Chicago, EUA), Resolute Integrity (Medtronic, Minneapolis, EUA), Supraflex (SMT, Mumbai, Índia), Orsiro (Biotronic, Berlim, Alemanha) e Ultimaster (Terumo, Tóquio, Japão). O número de pacientes tratados com cada tipo de SF, material de haste e espessura, droga antiproliferativa e tipo de polímero estão resumidos na Figura 1.

### Desfechos do estudo

Os desfechos clínicos analisados foram a ocorrência dos seguintes desfechos cardiovasculares maiores (MACCE) isolados ou combinados: novo infarto do miocárdio, trombose de stent, revascularização do vaso alvo, acidente vascular cerebral e óbito. O acompanhamento clínico foi realizado através de consulta ambulatorial ou contato telefônico.

### Análise estatística

As variáveis contínuas foram descritas como média e desvio padrão. As variáveis categóricas foram apresentadas como números absolutos e percentuais e comparadas usando o teste qui-quadrado ou teste exato de Fisher, quando apropriado. Os grupos de pacientes foram comparados usando o teste t de Student para amostras independentes para variáveis contínuas e qui-quadrado ou testes exatos de Fisher para variáveis categóricas. A normalidade da distribuição de cada variável foi avaliada pelo teste de Shapiro–Wilk.

Para limitar os vieses, foi usada a análise de pareamento por escore de propensão (PEP). Visto que as características basais dos dois grupos eram bastante diferentes e seu tamanho da amostra semelhante, selecionamos aleatoriamente 50% dos pacientes do grupo Inspiron® para reduzir a distância do escore de propensão e, portanto, reduzir grandes discrepâncias entre os grupos. A seleção aleatória foi realizada na plataforma Statistical Package for the Social Sciences (SPSS), com os seguintes comandos: selecionar casos ≥ amostra aleatória de casos ≥ 50% de todos os casos. A regressão logística foi realizada com Inspiron® como variável dependente e as seguintes variáveis independentes: idade, diabetes, creatinina na admissão, parada cardíaca pré-ICP e classificação de Killip. A validade da regressão logística foi avaliada usando o teste de Hosmer–Lemeshow. Subsequentemente, foi realizado PEP usando métodos do vizinho mais próximo, onde foram criados 2 grupos de 353 pacientes cada. A regressão de Cox para as taxas de eventos de acompanhamento de longo prazo de infarto do miocárdio, trombose de stent, revascularização, morte e MACCE foi calculada para população não pareada e grupos pareados.

Um modelo linear generalizado com regressão logística binária também foi realizado em pacientes gerais (e não em cima do PEP), e as mesmas variáveis do PEP foram incluídas como covariáveis em um modelo multivariado. Nosso objetivo foi mostrar resultados de dois modelos estatísticos diferentes comumente usados. Todos os testes de hipótese tiveram um nível de significância bilateral de 0,05. Todos os dados foram analisados usando SPSS, versão 17.0.

## Resultados

### Características clínicas basais

**Figura 2:**
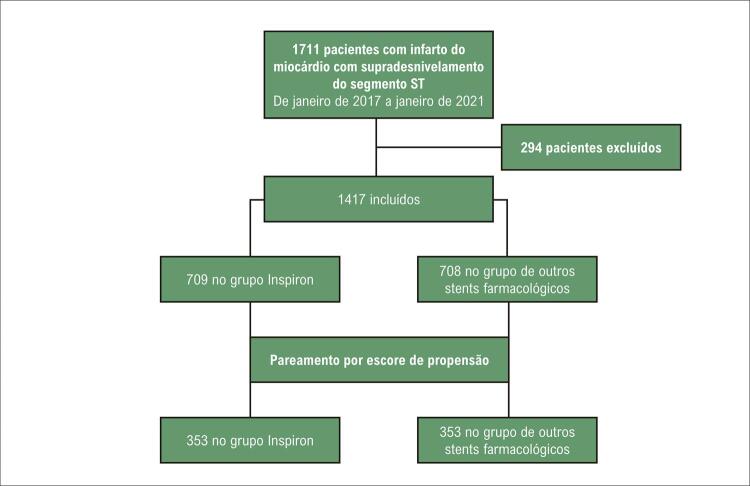
– Fluxograma do estudo.

De janeiro de 2017 a janeiro de 2021, 1.711 pacientes foram submetidos à ICP primária, e 1.417 pacientes preencheram nossos critérios de inclusão (709 no grupo Inspiron® e 708 nos outros DES de terceira geração) (Figura 2).

As taxas de hipertensão (60% versus 65%, p = 0,042), doença renal crônica (2,7% versus 6,6%, p < 0,001), choque cardiogênico na admissão (5,2% versus 9,5%, p = 0,002) e parada cardíaca (1,0 % versus 7,2%, p < 0,001) foram significativamente menores no grupo Inspiron®. O fluxo TIMI antes e após o procedimento foi diferente entre os grupos, e o comprimento total do stent foi menor no grupo Inspiron® (35 versus 41 mm, p < 0,001) (Tabela 1).

**Tabela 1:** – Características dos pacientes, apresentação clínica e aspectos do procedimento de acordo com o tipo de dispositivo

	Coorte não selecionada (sem pareamento)			Coorte pareada por escore de propensão		
	Inspiron®	Outro SF de terceira geração		Inspiron®	Outro SF de terceira geração	
	n = 709	n = 708	Valor p	n = 353	n = 353	Valor p
**Dados demográficos**						
Idade, anos	61,2 (±11,8)	61,3 (±12,4)	0,890	61,5 (±12,3)	61,3 (±12,4)	0,894
Sexo masculino	485 (68,4)	478 (67,5)	0,733	246 (69,7)	250 (70,8)	0,742
IMC, kg/m^2^	27,2 (4,4)	27,5 (4,7)	0,342	27,5 (4,7)	27,6 (4,9)	0,765
Hipertensão	428 (60,4)	465 (65,7)	0,042	215 (60,9)	224 (63,5)	0,485
Diabetes	208 (29,3)	237 (33,5)	0,097	101 (28,6)	100 (28,3)	0,934
História familiar de DAC	106 (15,0)	109 (15,4)	0,825	54 (15,3)	63 (17,8)	0,362
Doença renal crônica	19 (2,7)	47 (6,6)	<0,001	10 (2,8)	18 (5,1)	0,123
Tabagismo atual	293 (41,3)	266 (37,6)	0,348	68 (19,3)	67 (19,0)	0,526
Fibrilação atrial	5 (0,7)	11 (1,6)	0,141	1 (0,3)	5 (1,4)	0,101
Doença pulmonar	53 (7,5)	48 (6,8)	0,607	25 (7,2)	23 (6,5)	0,734
IM prévio	125 (17,6)	117 (16,5)	0,315	56 (15,9)	59 (16,7)	0,760
IC prévia	45 (6,3)	26 (3,7)	0,028	26 (7,4)	16 (4,5)	0,112
AVC prévio	38 (5,4)	45 (6,6)	0,317	18 (5,1)	23 (6,5)	0,421
**Avaliação de admissão**						
Choque cardiogênico	37 (5,2)	67 (9,5)	0,002	22 (6,2)	15 (4,2)	0,237
Parada cardíaca súbita	7 (1,0)	51 (7,2)	<0,001	2 (0,6)	1 (0,3)	0,563
IM anterior	343 (48,4)	344 (48,8)	0,915	176 (50)	165 (46,7)	0,387
Creatinina (g/dl)	1,06 (±0,59)	1,24 (±1,06)	<0,001	1,06 (±0,50)	1,09 (±0,75)	0,513
FEVE (%)	49,9 (±13,1)	49,9 (±12,7)	0,940	50,2 (±12,7)	50,1 (±12,4)	0,911
**Procedimento e acompanhamento hospitalar**						
Acesso radial	581 (83)	580 (83,9)	0,345	304 (86,1)	285 (80,7)	0,097
Lesão alvo			0,594			0,025
DAE	353 (50,1)	344 (48,7)		184 (52,4)	164 (46,6)	
ACD	265 (37,6)	257 (36,4)		134 (38,2)	129 (36,6)	
Circunflexo	72 (10,2)	64 (9,1)		24 (6,8)	34 (9,7)	
Doença principal esquerda	28 (3,9)	51 (7,2)	0,008	13 (3,7)	24 (6,8)	0,063
Doença de três vasos	116 (16,3)	136 (19,2)	0,920	65 (18,4)	66 (18,6)	0,974
Fluxo TIMI pré-procedimento						
0	490 (72,5)	536 (78,9)	<0,001	256 (72,5)	270 (79,6)	0,008
1	126 (18,6	120 (17,7)		68 (20,2)	67 (18,9)	
2	35 (5,2)	19 (2,8)		17 (5,1)	14 (3,9)	
3	25 (3,7)	4 (0,6)		12 (3,6)	2 (0,6)	
Aspiração de trombo	49 (7,0)	64 (9,1)	0,036	34 (9,6)	26 (7,4)	0,075
Número de stents	1 [1, 1]	1 [1, 2]	<0,001	2 [1, 3]	2 [1, 3]	0,253
Diâmetro médio do stent	3,0 (±0,5)	2,9 (±0,5)	0,075	3,0 (±0,5)	2,8 (±0,53)	0,094
Comprimento médio do stent	41 (±22)	35 (±18)	<0,001	41 (±23)	31 (±20)	<0,001
Fluxo TIMI pós-procedimento						
0	9 (1,3)	5 (0,7)	0,001	1 (0,3)	3 (0,9)	0,001
1	15 (2,1)	4 (0,6)		7 (2,0)	1 (0,3)	
2	52 (7,4)	26 (3,7)		32 (9,1)	15 (4,2)	
3	642 (90,5)	669 (95,0)		313 (88,9)	334 (94,6)	
Sucesso angiográfico	694 (99,0)	692 (99,0)	0,965	345 (97,7)	341 (96,6)	0,989

ACD: artéria coronária direita; AVC: acidente vascular cerebral; DAC: doença arterial coronariana; DAE: artéria descendente anterior esquerda; FEVE: fração de ejeção do ventrículo esquerdo; IC: insuficiência cardíaca; IM: infarto do miocárdio; IMC: índice de massa corporal; SF: stent farmacológico; TIMI: critérios Thrombolysis in Myocardial Infarction.

Após o PEP, a amostra do estudo foi composta por 706 pacientes (353 no Inspiron® grupo e 353 no grupo de outro SF de terceira geração). As diferenças nas características basais descritas acima perderam significância, com a exceção dos aspectos angiográficos. As características basais dos pacientes no grupo Inspiron® e no grupo de outro SF de terceira geração antes e depois do PEP estão resumidas na Tabela 1.

### Desfechos não ajustados

As taxas gerais de trombose de stent intra-hospitalar, acidente vascular cerebral, novo infarto do miocárdio e mortalidade foram de 0,7%, 1,3%, 1,4% e 7,5%, respectivamente. Os pacientes no grupo Inspiron® apresentaram menor mortalidade intra-hospitalar. As taxas de trombose de stent, acidente vascular cerebral e novo infarto do miocárdio foram semelhantes entre os grupos.

Após um acompanhamento médio de 17 meses, as taxas gerais de trombose de stent, acidente vascular cerebral, novo infarto do miocárdio, mortalidade e MACCE de longo prazo foram de 1,9%, 1,9%, 2,7%, 9,4% e 17,1%, respectivamente. Os pacientes no grupo Inspiron® tiveram menor infarto do miocárdio a longo prazo, acidente vascular cerebral, revascularização do vaso alvo, mortalidade e MACCE. A trombose de stent de longo prazo foi semelhante entre os grupos. Estes resultados estão resumidos na Tabela 2.

**Tabela 2:** – Acompanhamento de longo prazo de acordo com o dispositivo antes e depois do pareamento por escore de propensão

Coorte não selecionada				
	Inspiron®	Outro SF de terceira geração		
	n = 709	n = 708	OR (IC)	Valor p
Trombose de stent	12 (1,7)	15 (2,1)	0,79 (0,37 - 1,71)	0,568
Acidente vascular cerebral	3 (0,4)	24 (3,4)	0,12 (0,04 - 0,40)	<0,001
Infarto do miocárdio	12 (1,7)	26 (3,7)	0,45 (0,23 - 0,90)	0,022
Revascularização	15 (2,1)	30 (4,2)	0,49 (0,26 - 0,91)	0,024
Óbito	43 (6,1)	90 (12,7)	0,44 (0,30 - 0,65)	<0,001
MACCE	105 (14,8)	138 (19,5)	0,72 (0,54 - 0,95)	0,020

Coorte pareada por escore de propensão *				
	**Inspiron®**	**Outro SF de terceira geração**		
	**n = 353**	**n = 353**	**OR (CI)**	**Valor p**
Trombose de stent	5 (1,4)	5 (1,4)	1,00 (0,29 - 3,48)	1,000
Acidente vascular cerebral	1 (0,3)	9 (2,5)	0,11 (0,01 - 0,86)	0,011
Infarto do miocárdio	7 (2,0)	7 (2,0)	1,00 (0,35 - 2,88)	1,000
Revascularização	7 (2,0)	13 (3,7)	0,52 (0,21 - 1,34)	0,173
Óbito	23 (6,5)	31 (8,8)	0,72 (0,41 - 1,27)	0,257
MACCE	56 (15,9)	49 (13,9)	1,17 (0,77 - 1,77)	0,526

*Pareamento por escore de propensão ajustado por idade, diabetes, parada cardíaca pré-ICP, classificação de Killip, creatinina de admissão. IC: intervalo de confiança; MACCE: desfechos cardiovasculares, incluindo todos os desfechos acima; OR: odds ratio; SF: stent farmacológico.

### Pareamento por escore de propensão

Após PEP, as taxas gerais de trombose de stent intra-hospitalar, acidente vascular cerebral, novo infarto do miocárdio e mortalidade foram de 0,8%, 0,7%, 1,1% e 5,7%, respectivamente, e foram semelhantes entre os grupos.

As taxas gerais de trombose de stent, acidente vascular cerebral, novo infarto do miocárdio, revascularização do vaso alvo, mortalidade e MACCE de longo prazo foram de 1,4%, 1,4%, 2,0%, 2,6%, 7,6% e 14,9%, respectivamente. Os pacientes no grupo Inspiron® apresentaram taxas mais baixas de acidente vascular cerebral de longo prazo. Trombose de stent, revascularização do vaso alvo, mortalidade e MACCE de longo prazo foram semelhantes entre os grupos após acompanhamento a longo prazo. Estes resultados estão resumidos na Tabela 2.

### Análise multivariada da população geral

As mesmas variáveis incluídas no PEP foram usadas para criar o modelo multivariado na população geral. Os pacientes do grupo Inspiron® apresentaram taxas mais baixas de acidente vascular cerebral e revascularização do vaso alvo a longo prazo. Inspiron® não foi significativamente associado a taxas mais baixas de trombose de stent, infarto do miocárdio, morte ou MACCE de longo prazo (Tabela 3).

**Tabela 3:** – Análise multivariada dos desfechos de longo prazo usando a coorte não selecionada

	Trombose de stent		Acidente vascular cerebral		Revascularização	
	OR (IC 95%)	Valor p	OR (IC 95%)	Valor p	OR (IC 95%)	Valor p
Inspiron®	0,92 (0,42 - 2,04)	0,840	0,14 (0,04 - 0,46)	0,001	0,52 (0,27 - 0,98)	0,043
Idade	1,01 (0,98 - 1,05)	0,429	1,02 (0,99 - 1,06)	0,215	1,01 (0,98 - 1,03)	0,788
Diabetes	1,18 (0,53 - 2,63)	0,676	1,49 (0,68 - 3,25)	0,318	1,67 (0,91 - 3,07)	0,096
Parada cardíaca	1,96 (0,48 - 8,06)	0,349	1,08 (0,27 - 4,30)	0,914	1,04 (0,28 - 3,89)	0,957
Classificação de Killip	1,30 (0,88 - 1,93)	0,193	1,53 (1,06 - 2,20)	0,025	1,22 (0,88 - 1,69)	0,216
Creatinina de admissão	1,01 (0,67 - 1,50)	0,986	0,91 (0,59 - 1,41)	0,680	0,934 (0,65 - 1,35)	0,714

	**Infarto do miocárdio**		**Óbito**		**MACCE**	
	**OR (IC 95%)**	**Valor p**	**OR (IC 95%)**	**Valor p**	**OR (IC 95%)**	**Valor p**
Inspiron®	0,53 (0,26 - 1,08)	0,083	0,65 (0,42 - 1,01)	0,053	0,95 (0,70 - 1,29)	0,756
Idade	0,99 (0,97 - 1,02)	0,748	1,04 (1,03 - 1,07)	<0,001	1,02 (1,01 - 1,03)	0,002
Diabetes	1,93 (0,99 - 3,73)	0,051	2,02 (1,33 - 5,46)	0,001	1,41 (1,03 - 1,92)	0,029
Parada cardíaca	0,76 (0,20 - 2,85)	0,680	2,67 (1,31 - 5,46)	0,007	1,75 (0,92 - 3,34)	0,088
Classificação de Killip	1,47 (1,08 - 2,01)	0,013	2,27 (1,89 - 2,72)	<0,001	1,87 (1,61 - 2,18)	<0,001
Creatinina de admissão	1,25 (1,04 - 1,50)	0,015	1,43 (1,22 - 1,68)	<0,001	1,42 (1,20 - 1,68)	<0,001

IC: intervalo de confiança; MACCE: desfechos cardiovasculares, incluindo todos os desfechos acima; OR: odds ratio.

## Discussão

Em um registro contemporâneo do mundo real de pacientes com IAMCSST representativo da prática diária de hospitais terciários, um stent eluidor de sirolimus, de polímero biodegradável de cobalto-cromo, de hastes finas, demonstrou eficácia e segurança com baixa incidência de desfechos adversos em 12 meses. Embora as diferenças de linha de base fossem pronunciadas e os pacientes tratados com Inspiron® apresentassem um perfil de risco mais baixo, após PSM, Inspiron® não foi inferior em comparação com outros SF de segunda e terceira geração bem estabelecidos em relação a MACCE e seus componentes individuais.

Desde a introdução dos stents coronários no final de década de 80, houve contínuas melhorias técnicas e dos dispositivos visando reduzir os desfechos adversos relacionados tanto à apresentação clínica quanto às complicações relacionadas aos stents. Em comparação com os SF de primeira geração, os SF contemporâneos de segunda e terceira geração têm hastes mais finas, e a mudança na plataforma do stent de ligas de cromo de aço inoxidável (de 130–149 para 81–91 μm) reduziu o infarto do miocárdio do vaso alvo tardio e durante o procedimento.^4,5^ Hastes mais finas produzem menos lesões nos vasos, inflamação, proliferação neointimal e formação de trombos em comparação com stents com hastes mais espessas.^6,7^ Além disso, a espessura das hastes tem sido um elemento-chave no desenho do stent, pois hastes mais finas estão relacionadas a uma maior capacidade de entrega do stent. Por outro lado, hastes mais finas podem ter efeitos indesejáveis, como menor força radial e maior risco de deformação do stent ao negociar anatomias difíceis, o que destaca a importância de avaliar seus resultados em coortes contemporâneas de pacientes tratados na prática do mundo real.

Outra característica dos stents de terceira geração é a presença (em alguns deles) de polímero bioabsorvível. Isso possibilita a liberação controlada do fármaco e posterior dissolução do material polimérico, evitando estímulos para inflamação crônica e risco de posterior trombose do stent. Em um ensaio amplo do tipo “*all-comers*” comparando stents eluidores de sirolimus com polímero bioabsorvível e stents eluidores de everolimus com polímero durável, a ocorrência de eventos clínicos foi semelhante entre os grupos, embora o IAMCSST tenha sido a apresentação clínica em apenas 19% dos pacientes incluídos.^8^ Mais tarde, em um estudo randomizado envolvendo 1.334 pacientes (50% apresentando síndrome coronariana aguda), um stent eluidor de sirolimus, ultrafino e bioabsorvível teve menor falha na lesão alvo (6% versus 10%; intervalo de confiança 95% –6,84 a –0,29; p = 0,0399 ) e infarto do miocárdio no vaso alvo (5% versus 8%, p = 0,0155) em 12 meses em comparação com um stent eluidor de everolimus de polímero durável.^9^ Outro estudo comparando os mesmos SF acima, mas exclusivamente em pacientes com IAMCSST encontrou resultados semelhantes, com taxas mais baixas de falha da lesão alvo com um stent eluidor de sirolimus de polímero bioabsorvível.^10^ Essas diferenças, no entanto, podem ser causadas pela diferença na espessura da haste e não pela durabilidade do polímero, uma vez que o polímero do stent Orsiro® se degrada em um período de dois anos.

O polímero bioabsorvível isoladamente não garante a qualidade do SF. Metanálises anteriores indicaram um risco excessivo de eventos adversos com polímeros bioabsorvíveis em comparação com stents de polímeros duráveis, com alta heterogeneidade de dispositivos nos grupos de polímeros bioabsorvíveis.^11,12^ Por outro lado, uma metanálise mais recente envolvendo pacientes submetidos à ICP de artéria coronária principal esquerda desprotegida usando stents ultrafinos (hastes mais finas que 81 μm) mostrou resultados semelhantes em termos de MACCE com polímero bioabsorvível e stents de polímero durável, e diferenças na trombose de stent não foram evidentes entre os grupos.^13^ Em lesões de bifurcação tratadas com dois stents, no entanto, os pacientes tratados com SF de polímero biodegradável apresentaram melhor desfecho em termos de MACCE e revascularização do vaso alvo. Esses dados sugerem que evitar o estímulo inflamatório prolongado é especialmente importante em cenários mais trombogênicos, como síndromes coronarianas agudas e lesões de bifurcação. É notável que o Inspiron® suporta um acesso suave ao ramo lateral com seu design de célula aberta, e uma análise dedicada nesta configuração também é merecida.

Estudos avaliando o stent Inspiron® relataram propriedades promissoras de cicatrização dos vasos, com pouca hiperplasia neointimal por ultrassom intravascular (percentual de obstrução por hiperplasia neointimal de 4,9% ± 4,1%) e altas taxas de cobertura das hastes por tomografia de coerência óptica (99,49% ± 1,01%).^14^ Embora o Inspiron® tenha demonstrado ser seguro em um ensaio clínico randomizado prévio com uma população “*all-comers*” com um longo acompanhamento,^3^ o uso generalizado de dispositivos mais novos pode levar tempo, especialmente em pacientes e anatomias de risco elevados. Em nosso estudo, o risco basal da população Inspiron® foi claramente menor, e uma das hipóteses é que os operadores tendem a escolher dispositivos bem estabelecidos em casos mais complexos. O grupo Inspiron® apresentou taxas mais baixas de acidente vascular cerebral de longo prazo, mesmo após o PEP, possivelmente devido a outras variáveis de confusão não incluídas no modelo.

O presente estudo tem limitações, em primeiro lugar, as limitações inerentes aos estudos observacionais, onde a escolha do tratamento foi baseada na preferência do operador. O viés de seleção foi altamente provável, embora a análise estatística possa ter atenuado esse problema. Em segundo lugar, a análise retrospectiva pode ter influenciado a qualidade e a consistência dos dados coletados. No entanto, este foi um registro representativo de IAMCSST de dois centros com critérios de inclusão amplos e dados clínicos altamente aplicáveis.

## Conclusões

Nossos achados sustentam que o Inspiron® é seguro e eficaz em pacientes com IAMCSST, com desfechos semelhantes em comparação com SF de terceira geração bem estabelecidos no tratamento com ICP primária em acompanhamento de curto e longo prazo.
