# Supplementary material for: Avaliação no Mundo Real de um Stent Eluidor de Sirolimus de Hastes Ultrafinas em Pacientes com Infarto do Miocárdio com Supradesnivelamento do Segmento ST Submetidos à Intervenção Coronária Percutânea Primária (Registro INSTEMI)
Source: Arq Bras Cardiol. 2023 May 18;120(6):e20220594. [Article in Portuguese] doi: 10.36660/abc.20220594 (PMC10228630; doi:10.36660/abc.20220594)
Supplement: Translation

# Translation: Real-World Assessment of an Ultrathin Strut, Sirolimus-Eluting Stent in Patients with ST-Elevation Myocardial Infarction Submitted to Primary Percutaneous Coronary Intervention (INSTEMI Registry)

**Authors:** Gustavo N. Araujo, Guilherme P. Machado, Marcia Moura, Anderson D. Silveira, Luiz Carlos Bergoli, Felipe Costa Fuchs, Sandro Cadaval Gonçalves, Rodrigo Vugman Wainstein, Pedro A. Lemos, Alexandre S. Quadros, Marco V. Wainstein

**Affiliations:** 1 Brasil Imperial Hospital de Caridade, Florianópolis, SC – Brasil; 2 Brasil Instituto de Cardiologia de Santa Catarina, São José, SC – Brasil; 3 Brasil Hospital de Clínicas de Porto Alegre, Porto Alegre, RS – Brasil; 4 Brasil Instituto de Cardiologia do Rio Grande do Sul, Porto Alegre, RS – Brasil; 5 Brasil Hospital Moinhos de Vento, Porto Alegre, RS – Brasil; 6 Brasil Universidade Federal do Rio Grande do Sul, Porto Alegre, RS – Brasil; 7 Brasil Hospital Israelita Albert Einstein, São Paulo, SP – Brasil; 8 Brasil Instituto do Coração (InCor), Universidade de São Paulo, São Paulo, SP – Brasil

**Keywords:** Myocardial Infarction, Angioplasty, Drug-Eluting Stents

## Abstract

**Background:**

The current gold standard of coronary drug-eluting stents (DES) consists of metal alloys with thinner struts and bioresorbable polymers.

**Objectives:**

Our aim was to compare an ultrathin strut, sirolimus-eluting stent (Inspiron®) with other third-generation DES platforms in patients with ST-elevation myocardial infarction (STEMI) submitted to primary percutaneous coronary intervention (PCI).

**Methods:**

We analyzed data from a STEMI multicenter registry from reference centers in the South Region of Brazil. All patients were submitted to primary PCI, either with Inspiron® or other second- or third-generation DES. Propensity score matching (PSM) was computed to generate similar groups (Inspiron® versus other stents) in relation to clinical and procedural characteristics. All hypothesis tests had a two-sided significance level of 0.05.

**Results:**

From January 2017 to January 2021, 1711 patients underwent primary PCI, and 1417 patients met our entry criteria (709 patients in the Inspiron® group and 708 patients in the other second- or third-generation DES group). After PSM, the study sample was comprised of 706 patients (353 patients in the Inspiron® group and 353 patients in the other the other second- or third-generation DES group). The rates of target vessel revascularization (OR 0.52, CI 0.21 – 1.34, p = 0.173), stent thrombosis (OR 1.00, CI 0.29 – 3.48, p = 1.000), mortality (HR 0.724, CI 0.41 – 1.27, p = 0.257), and major cardiovascular outcomes (OR 1.170, CI 0.77 – 1.77, p = 0.526) were similar between groups after a median follow-up of 17 months.

**Conclusion:**

Our findings show that Inspiron® was effective and safe when compared to other second- or third-generation DES in a contemporary cohort of real-world STEMI patients submitted to primary PCI.

**Central Illustration:** : Real-World Assessment of an Ultrathin Strut, Sirolimus-Eluting Stent in Patients with ST-Elevation Myocardial Infarction Submitted to Primary Percutaneous Coronary Intervention (INSTEMI Registry)

## Introduction

Coronary drug-eluting stents (DES) are continuously evolving, and newer devices should present safety and efficacy in order to be used in daily practice. ST-elevation myocardial infarction (STEMI) is probably the most challenging clinical scenario to compare newer DES with established ones, because of higher in-hospital and long-term risks of stent thrombosis, recurrent myocardial infarction, and death.

The Inspiron® sirolimus-eluting stent (Scitech Medical^TM^, Goiânia, Brazil) uses an ultrathin L-605 cobalt-chromium alloy with a 75 μm rod thickness platform coated with a biodegradable abluminal polymer. Registries of all-comers population demonstrated safety and excellent performance, with a low rate of adverse cardiac events.^1,2^ In a randomized clinical trial comparing Inspiron® with Biomatrix Flex® biolimus-eluting stent, there was no difference in outcomes in patients undergoing elective or urgent percutaneous coronary intervention (PCI) after a five-year follow-up. Also, there was no stent thrombosis in patients treated with Inspiron® during the study period.^3^

With the widespread use of the device, data from a greater number of patients in a high-risk scenario are needed. Our aim was to compare the Inspiron® sirolimus-eluting stent with other safe, well studied and established third-generation DES platforms in patients with STEMI submitted to primary PCI.

## Methods

### Study design and patient selection

This is a prospective registry, in which we included consecutive patients admitted with STEMI and treated with primary PCI using second- and third-generation DES in two tertiary hospitals (Hospital de Clinicas de Porto Alegre, a general hospital, and Instituto de Cardiologia do Rio Grande do Sul, a cardiology center) in the South Region of Brazil between the years of 2017 and 2021. This specific analysis was conducted retrospectively; that is, it had not been pre-defined at the time registration was started. STEMI was defined as typical chest pain at rest associated with ST-segment elevation of at least 1 mm in two contiguous leads in the frontal plane or 2 mm in the horizontal plane, or typical pain at rest in patients with a new, or presumably new, left bundle-branch block. Exclusion criteria were absence of second- and third-generation DES use and lack of follow-up. This study was approved by the Institutional Research and Ethics Committee of both institutions, and informed consent was obtained from all patients. The data were prospectively recorded in appropriate forms, stored in electronic spreadsheets and later collected from the database.

### Procedural aspects

Blood samples were collected by venipuncture before the procedure, as part of routine patient care. All patients were pre-treated with a loading dose of acetylsalicylic acid (300 mg) and clopidogrel (300 to 600 mg), and unfractioned heparin was used during the procedure (70 to 100 IU/kg). PCI technical strategies and stent selection were performed according to the operator’s choice. Coronary flow before and after the procedure was assessed and described according to the Thrombolysis in Myocardial Infarction (TIMI) criteria. Anticoagulants were suspended after the end of procedure, and dual antiplatelet therapy duration was recommended at the cardiologist’s discretion. Primary PCI success was defined as achievement of TIMI flow 2 or 3 and vessel patency with residual stenosis < 30%.

### Stents

All patients included received Inspiron® or a second- or third-generation DES. The decision to implant which type of stent was based on operator discretion and center availability. Besides Inspiron®, the other platforms used were Xience (Abbott laboratories, Chicago, USA), Resolute Integrity (Medtronic, Minneapolis, USA), Supraflex (SMT, Mumbai, India), Orsiro (Biotronic, Berlin, Germany), and Ultimaster (Terumo, Tokyo, Japan). The number of patients treated with each type of DES, strut material and thickness, antiproliferative drug, and polymer type are summarized in Figure 1.

**Figure 1:** – Characteristics of the drug-eluting stents used in the study.

### Study outcomes

The clinical outcomes analyzed were the occurrence of the following isolated or combined major cardiovascular outcomes (MACCE): a new myocardial infarction, stent thrombosis, target vessel revascularization, stroke, and death. Clinical follow-up was carried out through outpatient consultation or telephone contact.

### Statistical analysis

Continuous variables were described as mean and standard deviation. Categorical variables were presented as absolute and percentage numbers and compared using the chi-square test or Fisher’s exact test, when appropriate. Patient groups were compared using Student’s t test for independent samples for continuous variables and chi-square or Fisher’s exact tests for categorical variables. The normality of the distribution of each variable was assessed by the Shapiro–Wilk test.

To limit biases, propensity score matching (PSM) analysis was used. Because the baseline characteristics of the two groups were quite different and their sample size were similar, we randomly selected 50% of the Inspiron® patients in order to reduce the propensity score distance and therefore reduce large score discrepancies between groups. The random selection was performed in the Statistical Package for the Social Sciences (SPSS) platform, with the following commands: select cases ≥ random sample of cases ≥ 50% of all the cases. The logistic regression was performed with Inspiron® as a dependent variable and the following as independent variables: age, diabetes, admission creatinine, pre-PCI cardiac arrest, and Killip classification. The validity of logistic regression was assessed using the Hosmer–Lemeshow test. Subsequently, PSM was performed using nearest neighbor methods, where 2 groups of 353 patients each were created. Cox regression for long-term follow-up event rates of myocardial infarction, stent thrombosis, revascularization, death, and MACCE was calculated for unmatched population and matched groups.

A generalized linear model with binary logistic regression was also performed in overall patients (and not on top of PSM), and the same variables of PSM were included as covariates in a multivariate model. Our objective was to show results of two different statistical models commonly used. All hypothesis tests had a two-sided significance level of 0.05 All data were analyzed using SPSS, version 17.0.

## Results

### Baseline clinical characteristics

From January 2017 to January 2021, 1711 patients underwent primary PCI and 1417 patients met our entry criteria (709 in the Inspiron® group and 708 in the other third-generation DES group) (Figure 2).

**Figure 2:** – Study flowchart.

Rates of hypertension (60% versus 65%, p = 0.042), chronic kidney disease (2.7% versus 6.6%, p < 0.001), admission cardiogenic shock (5.2% versus 9.5%, p = 0.002), and cardiac arrest (1.0% versus 7.2%, p < 0.001) were significantly lower in the Inspiron® group. Pre- and post-procedural TIMI flow distribution was different among groups, and total stent length was shorter in the Inspiron® group (35 versus 41 mm, p < 0.001) (Table 1).

**Table 1:** – Patients characteristics, clinical presentation, and procedural aspects according to device type

	Unselected cohort (non-matched)			Propensity score-matched cohort		
	Inspiron®	Other third-generation DES		Inspiron®	Other third-generation DES	
	n = 709	n = 708	p value	n = 353	n = 353	p value
**Demographic data**						
Age, years	61.2 (±11.8)	61.3 (±12.4)	0.890	61.5 (±12.3)	61.3 (±12.4)	0.894
Male	485 (68.4)	478 (67.5)	0.733	246 (69.7)	250 (70.8)	0.742
BMI, kg/m^2^	27.2 (4.4)	27.5 (4.7)	0.342	27.5 (4.7)	27.6 (4.9)	0.765
Hypertension	428 (60.4)	465 (65.7)	0.042	215 (60.9)	224 (63.5)	0.485
Diabetes	208 (29.3)	237 (33.5)	0.097	101 (28.6)	100 (28.3)	0.934
Family history of CAD	106 (15.0)	109 (15.4)	0.825	54 (15.3)	63 (17.8)	0.362
Chronic kidney disease	19 (2.7)	47 (6.6)	<0.001	10 (2.8)	18 (5.1)	0.123
Current tobacco use	293 (41.3)	266 (37.6)	0.348	68 (19.3)	67 (19.0)	0.526
Atrial fibrillation	5 (0.7)	11 (1.6)	0.141	1 (0.3)	5 (1.4)	0.101
Lung disease	53 (7.5)	48 (6.8)	0.607	25 (7.2)	23 (6.5)	0.734
Previous MI	125 (17.6)	117 (16.5)	0.315	56 (15.9)	59 (16.7)	0.760
Previous HF	45 (6.3)	26 (3.7)	0.028	26 (7.4)	16 (4.5)	0.112
Previous stroke	38 (5.4)	45 (6.6)	0.317	18 (5.1)	23 (6.5)	0.421
**Admission evaluation**						
Cardiogenic shock	37 (5.2)	67 (9.5)	0.002	22 (6.2)	15 (4.2)	0.237
Sudden cardiac arrest	7 (1.0)	51 (7.2)	<0.001	2 (0.6)	1 (0.3)	0.563
Anterior MI	343 (48.4)	344 (48.8)	0.915	176 (50)	165 (46.7)	0.387
Creatinine (g/dl)	1.06 (±0.59)	1.24 (±1.06)	<0.001	1.06 (±0.50)	1.09 (±0.75)	0.513
LVEF (%)	49.9 (±13.1)	49.9 (±12.7)	0.940	50.2 (±12.7)	50.1 (±12.4)	0.911
**Procedure and in-hospital follow-up**						
Radial access	581 (83)	580 (83.9)	0.345	304 (86.1)	285 (80.7)	0.097
Target lesion			0.594			0.025
LAD	353 (50.1)	344 (48.7)		184 (52.4)	164 (46.6)	
RCA	265 (37.6)	257 (36.4)		134 (38.2)	129 (36.6)	
Circumflex	72 (10.2)	64 (9.1)		24 (6.8)	34 (9.7)	
Left main disease	28 (3.9)	51 (7.2)	0.008	13 (3.7)	24 (6.8)	0.063
Three-vessel disease	116 (16.3)	136 (19.2)	0.920	65 (18.4)	66 (18.6)	0.974
Pre-procedure TIMI flow						
0	490 (72.5)	536 (78.9)	<0.001	256 (72.5)	270 (79.6)	0.008
1	126 (18.6	120 (17.7)		68 (20.2)	67 (18.9)	
2	35 (5.2)	19 (2.8)		17 (5.1)	14 (3.9)	
3	25 (3.7)	4 (0.6)		12 (3.6)	2 (0.6)	
Thrombus aspiration	49 (7.0)	64 (9.1)	0.036	34 (9.6)	26 (7.4)	0.075
Number of stents	1 [1, 1]	1 [1, 2]	<0.001	2 [1, 3]	2 [1, 3]	0.253
Mean stent diameter	3.0 (±0.5)	2.9 (±0.5)	0.075	3.0 (±0.5)	2.8 (±0.53)	0.094
Mean stent length	41 (±22)	35 (±18)	<0.001	41 (±23)	31 (±20)	<0.001
Post-procedure TIMI flow						
0	9 (1.3)	5 (0.7)	0.001	1 (0.3)	3 (0.9)	0.001
1	15 (2.1)	4 (0.6)		7 (2.0)	1 (0.3)	
2	52 (7.4)	26 (3.7)		32 (9.1)	15 (4.2)	
3	642 (90.5)	669 (95.0)		313 (88.9)	334 (94.6)	
Angiographic success	694 (99.0)	692 (99.0)	0.965	345 (97.7)	341 (96.6)	0.989

BMI: body mass index; CAD: coronary artery disease; DES: drug-eluting stent; HF: heart failure; LAD: left anterior descending artery; LVEF: left ventricular ejection fraction; MI: myocardial infarction; RCA: right coronary artery; TIMI: Thrombolysis in Myocardial Infarction criteria.

After PSM, the study sample comprised 706 patients (353 in the Inspiron® group and 353 in the other third-generation DES group). Differences in baseline characteristics described above have lost significance, except for angiographic aspects. Baseline characteristics of patients in the Inspiron® and other third-generation DES groups before and after PSM are summarized in Table 1.

### Unadjusted outcomes

Overall in-hospital stent thrombosis, stroke, new myocardial infarction, and mortality were 0.7%, 1.3%, 1.4%, and 7.5%, respectively. Patients in the Inspiron® group had lower in-hospital mortality. Rates of stent thrombosis, stroke, and new myocardial infarction were similar between groups.

After a median follow-up of 17 months, overall long-term stent thrombosis, stroke, new myocardial infarction, mortality, and MACCE were 1.9%, 1.9%, 2.7%, 9.4%, and 17.1%, respectively. Patients in the Inspiron® group had lower long-term myocardial infarction, stroke, target vessel revascularization, mortality, and MACCE. Long-term stent thrombosis was similar between groups. These findings are summarized in Table 2.

**Table 2:** – Long-term follow-up according to device before and after propensity score matching

Unselected cohort				
	Inspiron®	Other third-generation DES		
	n = 709	n = 708	OR (CI)	p value
Stent thrombosis	12 (1.7)	15 (2.1)	0.79 (0.37 - 1.71)	0.568
Stroke	3 (0.4)	24 (3.4)	0.12 (0.04 - 0.40)	<0.001
Myocardial infarction	12 (1.7)	26 (3.7)	0.45 (0.23 - 0.90)	0.022
Revascularization	15 (2.1)	30 (4,2)	0.49 (0.26 - 0.91)	0.024
Death	43 (6.1)	90 (12.7)	0.44 (0.30 - 0.65)	<0.001
MACCE	105 (14.8)	138 (19.5)	0.72 (0.54 - 0.95)	0.020

Propensity score-matched cohort*				
	**Inspiron®**	**Other third-generation DES**		
	**n = 353**	**n = 353**	**OR (CI)**	**p value**
Stent thrombosis	5 (1.4)	5 (1.4)	1.00 (0.29 - 3.48)	1.000
Stroke	1 (0.3)	9 (2.5)	0.11 (0.01 - 0.86)	0.011
Myocardial infarction	7 (2.0)	7 (2.0)	1.00 (0.35 - 2.88)	1.000
Revascularization	7 (2.0)	13 (3.7)	0.52 (0.21 - 1.34)	0.173
Death	23 (6.5)	31 (8.8)	0.72 (0.41 - 1.27)	0.257
MACCE	56 (15.9)	49 (13.9)	1.17 (0.77 - 1.77)	0.526

*Propensity score matching adjusted for age, diabetes, pre-PCI cardiac arrest, Killip classification, admission creatinine. CI: confidence interval; DES: drug-eluting stent; MACCE: major cardiovascular outcomes including all the outcomes above; OR: odds ratio.

### Propensity score matching

After PSM, the rates of overall in-hospital stent thrombosis, stroke, new myocardial infarction, and mortality were 0.8%, 0.7%, 1.1%, and 5.7%, respectively, and they were similar between groups.

Rates of overall long-term stent thrombosis, stroke, new myocardial infarction, target vessel revascularization, mortality, and MACCE were 1.4%, 1.4%, 2.0%, 2.6%, 7.6%, and 14.9%, respectively. Patients in the Inspiron® group had lower rates of long-term stroke. Long-term stent thrombosis, target vessel revascularization, mortality, and MACCE were similar between groups after long-term follow up. These findings are summarized in Table 2.

### Multivariate analysis of overall population

The same variables included in the PSM were used to create the multivariate model in overall population. Patients in the Inspiron® group had lower rates of long-term stroke and target vessel revascularization. Inspiron® was not significantly associated with lower rates of long-term stent thrombosis, myocardial infarction, death, or MACCE (Table 3).

**Table 3:** – Multivariate analysis of long-term outcomes using the unselected cohort

	Stent Thrombosis		Stroke		Revascularization	
	OR (95% CI)	p value	OR (95% CI)	p value	OR (95% CI)	p value
Inspiron®	0.92 (0.42 - 2.04)	0.840	0.14 (0.04 - 0.46)	0.001	0.52 (0.27 - 0.98)	0.043
Age	1.01 (0.98 - 1.05)	0.429	1.02 (0.99 - 1.06)	0.215	1.01 (0.98 - 1.03)	0.788
Diabetes	1.18 (0.53 - 2.63)	0.676	1.49 (0.68 - 3.25)	0.318	1.67 (0.91 - 3.07)	0.096
Cardiac arrest	1.96 (0.48 - 8.06)	0.349	1.08 (0.27 - 4.30)	0.914	1.04 (0.28 - 3.89)	0.957
Killip classification	1.30 (0.88 - 1.93)	0.193	1.53 (1.06 - 2.20)	0.025	1.22 (0.88 - 1.69)	0.216
Admission creatinine	1.01 (0.67 - 1.50)	0.986	0.91 (0.59 - 1.41)	0.680	0.934 (0.65 - 1.35)	0.714

	**Myocardial Infarction**		**Death**		**MACCE**	
	**OR (95% CI)**	**p value**	**OR (95% CI)**	**p value**	**OR (95% CI)**	**p value**
Inspiron®	0.53 (0.26 - 1.08)	0.083	0.65 (0.42 - 1.01)	0.053	0.95 (0.70 - 1.29)	0.756
Age	0.99 (0.97 - 1.02)	0.748	1.04 (1.03 - 1.07)	<0.001	1.02 (1.01 - 1.03)	0.002
Diabetes	1.93 (0.99 - 3.73)	0.051	2.02 (1.33 - 5.46)	0.001	1.41 (1.03 - 1.92)	0.029
Cardiac arrest	0.76 (0.20 - 2.85)	0.680	2.67 (1.31 - 5.46)	0.007	1.75 (0.92 - 3.34)	0.088
Killip classification	1.47 (1.08 - 2.01)	0.013	2.27 (1.89 - 2.72)	<0.001	1.87 (1.61 - 2.18)	<0.001
Admission creatinine	1.25 (1.04 - 1.50)	0.015	1.43 (1.22 - 1.68)	<0.001	1.42 (1.20 - 1.68)	<0.001

CI: confidence interval; MACCE: major cardiovascular outcomes including all the outcomes above; OR: odds ratio.

## Discussion

In a contemporary, real-world registry of STEMI patients representative of the daily practice of tertiary hospitals, a thin strut, cobalt-chromium, biodegradable polymer sirolimus-eluting stent demonstrated effectiveness and safety with a low incidence of adverse outcomes at 12 months. Although baseline differences were pronounced and patients treated with Inspiron® displayed a lower risk profile, after PSM, Inspiron® was not inferior compared to other well established second- and third-generation DES regarding MACCE and its individual components.

Since the introduction of coronary stents in late 80s, there have been continuing technical and device improvements aimed at reducing adverse outcomes related to both clinical presentation and stent-related complications. Compared to first-generation DES, contemporary second- and third-generation DES have thinner struts, and the change in stent platform from stainless steel chromium alloys (130–149 to 81–91 μm) reduced both procedural and late target vessel myocardial infarction.^4,5^ Thinner struts produce less vessel injury, inflammation, neointimal proliferation, and thrombus formation compared with thicker strut stents.^6,7^ Moreover, strut thickness has been a key element in stent design, as thinner struts are related to a greater stent deliverability. On the other hand, thinner struts may have undesirable effects, such as lower radial force and higher risk of stent deformation when negotiating difficult anatomies, which highlights the importance of assessing its results on contemporary cohorts of patients treated in real-world practice.

Another characteristic of third-generation stents is the presence (in some of them) of bioresorbable polymer. It enables controlled drug release and subsequent dissolution of the polymer material, avoiding stimulus for chronic inflammation risk of further stent thrombosis. In a large, all-comers trial comparing bioresorbable polymer sirolimus-eluting stents and durable polymer everolimus-eluting stents, the occurrence of clinical events was similar between groups, although STEMI was the clinical presentation in only 19% of included patients.^8^ Later, in a randomized trial involving 1334 patients (50% presenting with acute coronary syndrome), an ultrathin, bioresorbable polymer sirolimus-eluting stent had lower target lesion failure (6% versus 10%, 95% CI –6.84 to –0.29, p = 0.0399) and target vessel myocardial infarction (5% versus 8%, p = 0.0155) at 12 months compared to a durable polymer everolimus-eluting stent.^9^ Another study comparing the same DES above, but exclusively in patients with STEMI found similar results, with lower rates of target lesion failure with a bioresorbable polymer sirolimus-eluting stent.^10^ These differences, however, may be driven by the difference in strut thickness rather than polymer durability, since the Orsiro® stent polymer degrades over a two-year span.

Bioresorbable polymer alone does not guarantee the quality of the DES. Previous meta-analyses have indicated an excess risk of adverse events with bioresorbable polymer compared with durable polymer stents, with a high heterogeneity of devices in the bioresorbable polymer groups.^11,12^ Conversely, a more recent meta-analysis involving patients submitted to PCI of unprotected left main coronary artery using ultrathin stents (struts thinner than 81 μm) showed similar results in terms of MACCE with bioresorbable polymer and durable polymer stents, and no differences in stent thrombosis were evident between groups.^13^ In bifurcation lesions treated with two stents, however, patients treated with biodegradable polymer DES showed a better outcome in terms of MACCE and target-vessel revascularization. These data suggest that avoiding prolonged inflammatory stimulus is especially important in more thrombogenic settings, such as acute coronary syndromes and bifurcation lesions. It is noteworthy that Inspiron® supports a smooth side branch access with its open-cell design, and dedicated analysis in this setting is also warranted.

Studies evaluating Inspiron® stent have reported reassuring vessel healing properties, with very little neointimal hyperplasia either by intravascular ultrasound (percent of neointimal hyperplasia obstruction of 4.9% ± 4.1%) and high rates of strut coverage by optical coherence tomography (99.49% ± 1.01%).^14^ Although Inspiron® was demonstrated safe in a previous randomized clinical trial with an all-comers population with a long follow-up,^3^ widespread use of newer devices may take time, especially in higher risk patients and anatomy. In our study, baseline risk of the Inspiron® population was clearly lower, and one of the hypotheses is that operators tend to choose well established devices in more complex cases. The Inspiron® group had lower rates of long-term stroke, even after PSM, possibly because of other confounding variables not included in the model.

This study has limitations, first, the limitations that are inherent in observational studies, where choice of treatment was based on the operator’s preference. Selection bias was highly probable, although statistical analysis may have mitigated this issue. Second, the retrospective analysis may have influenced the quality and consistency of the data collected. However, this was a representative two-center STEMI registry with broad inclusion criteria and highly applicable clinical data.

## Conclusions

Our findings support that Inspiron® is safe and effective in patients with STEMI, with similar outcomes compared to well established third-generation DES in treatment with primary PCI at short- and long-term follow-up.
